# A PRISMA-compliant meta-analysis of MDR1 polymorphisms and idiopathic nephrotic syndrome: Susceptibility and steroid responsiveness: Erratum

**DOI:** 10.1097/MD.0000000000007453

**Published:** 2017-06-30

In the article, “A PRISMA-compliant meta-analysis of MDR1 polymorphisms and idiopathic nephrotic syndrome: Susceptibility and steroid responsiveness”,^[1]^ which appeared in Volume 96, Issue 24 of *Medicine*, the caption of Table 2 should have appeared as “^∗^The star system from Newcastle-Ottawa Scale, a maximum of 1 ‘^∗^’ was awarded for each item from the selection and exposure, a maximum of 2 ‘^∗^’ could be assigned for comparability.”
